# German guideline on neurosyphilis

**DOI:** 10.1186/s42466-026-00525-0

**Published:** 2026-08-21

**Authors:** Matthias Klein, Klemens Angstwurm, Stefan Esser, Juliane Fazio, Katrin Hahn, Simone Scheithauer, Anja Potthoff, Lara Chilver-Stainer, Jörg Weber, Ricardo Niklas Werner, Brigitte Wildmann, Matthias Maschke

**Affiliations:** 1 Department of Neurology, Ludwig-Maximilians University Munich, Marchioninistr. 15, Munich, 81377 Germany; 2 German Society of Neurology (DGN), Berlin, Germany; 3 Department of Neurology, University of Regensburg, Regensburg, Germany; 4 German Neurocritical Care Society (DGNI), Jena, Germany; 5 Department of Dermatology, University of Duisburg-Essen, Essen, Germany; 6 German AIDS Society (DAIG), Decentralized, Germany; 7 Laboratory Krone, Bad Salzuflen, Germany; 8 Department of Neurology Berlin, Charite University Medicine Berlin, Berlin, Germany; 9 Department of Infection Control and Infectious Diseases, University Medical Center Goettingen, Göttingen, Germany; 10 German Society for Hygiene and Microbiology (DGHM), Hannover, Germany; 11 Catholic Hospital Bochum, Bochum, Germany; 12 German STI – Society (DSTIG), Bochum, Germany; 13 Department of Neurology, Inselspital, Bern University Hospital, University of Bern, Switzerland (CH), Bern, Germany; 14 Swiss Neurological Society, Basel, Switzerland; 15 Department of Neurology, Hospital of Klagenfurt, Klagenfurt am Wörthersee, Austria; 16 Austrian Society of Neurology (ÖGN), Vienna, Austria; 17 Department of Dermatology, Venereology and Allergology, Charite Universitätsmedizin Berlin, Berlin, Germany; 18 German Society of Dermatology (DDG), Berlin, Germany; 19 Department of Neurology, Heidelberg University, Heidelberg, Germany; 20 German Society for CSF diagnostics and clinical neurochemistry (DGLN), Ulm, Germany; 21 Department of Neurology, Hospital Trier, Trier, Germany

## Abstract

Neurosyphilis is a severe disease which can manifest with various clinical signs. The diagnosis can be challenging. Patients with positive syphilis serology and neurological or psychiatric symptoms should undergo cerebrospinal fluid (CSF) examination. The definite diagnosis is based on CSF findings, including evidence of inflammation, intrathecal production of treponemal antibodies (plus reactive non-treponema specific tests in the CSF). However, these criteria are not always fulfilled and a diagnosis of probable neurosyphilis may be established using less stringent diagnostic criteria. Treatment with intravenous penicillin or ceftriaxone is recommended for neurosyphilis. Here, a short version of the recommendations of the German guidelines on neurosyphilis that were published in 2026 is presented.

## Introduction

Syphilis is caused by *Treponema pallidum*, a Gram-negative bacterium belonging to the family *Spirochaetaceae*. *Treponema pallidum* is transmitted through sexual contact in most cases. Possible but rare routes of transmission include blood transfusion, organ transplantation, and vertical transmission (during pregnancy, peripartum, or postpartum). Approximately one third of infected individuals develop clinical manifestations. Syphilis is an infectious disease that classically progresses through several stages [1, 2]. Following a localized infection at the site of entry (primary syphilis), a chronic relapsing disease with various manifestations develops (secondary syphilis).

The World Health Organization (WHO) defines early syphilis as symptomatic disease lasting no longer than two years whereas the European guidelines, and the guidelines of the Centers for Disease Control and Prevention (CDC) and the German AWMF S2k Guideline “Diagnosis and Treatment of Syphilis” define late syphilis as infection persisting after at least one year [3, 4]. The principal manifestations of early neurosyphilis are symptomatic meningitis and, usually later, meningovascular neurosyphilis. Syphilitic gummas may also develop. Late neurosyphilis typically presents as paralytic neurosyphilis (synonyms: progressive paralysis, dementia paralytica, general paresis) or tabetic neurosyphilis (synonym: tabes dorsalis). In addition, atypical forms.

This article summarizes the key recommendations of the updated German guideline on diagnosis and treatment of neurosyphilis which was published in 2026 [5]. Recommendations are given on how the diagnosis of neurosyphilis is made in HIV-positive and HIV-negative patients. Finally, recommendations on treatment strategies are pointed out. The original German version of the guideline can be found at www.dgn.org/leitlinie/neurosyphilis. AWMF-registry number: 030/101 [5].

### Epidemiology

According to the WHO, approximately 7.1 million people worldwide develop syphilis each year [6]. The global prevalence is estimated at 22.3 million known cases [7]. In Germany, the incidence of syphilis, based on data reported to the Robert Koch Institute (RKI), declined for many years and reached its lowest recorded level at the end of the 1990s, with 1.4 reported cases per 100,000 inhabitants [8]. Since 2010, however, the number of new infections has steadily increased [9].

Among men who have sex with men (MSM), who accounted for 74,6% of all syphilis cases in Germany in 2024, 35.6% of reported syphilis cases involved HIV co-infection, whereas this proportion was substantially lower (7.9%) among cases with a probable heterosexual route of transmission [9]. HIV infection appears to increase the risk of developing neurosyphilis [10]. HIV-positive patients with early or late syphilis exhibit higher rates of neurosyphilis compared with patients without HIV (1.2% vs. 0.7%) [11]. Overall, the risk appears particularly elevated for early manifestations of neurosyphilis in HIV-positive patients [12, 13]. Other co-infections frequently diagnosed alongside syphilis in Germany include chlamydia, gonorrhea, hepatitis B, and hepatitis C [14].

At the time of diagnosis, 25.1% of patients in Germany were in the primary stage of syphilis, 17.1% in the secondary stage, 1.5% in the tertiary stage, 24.0% in the early latent phase, 2.1% in the late latent phase, and 27.5% had syphilis of unknown duration; the remainder lacked stage information [9].

## Clinical manifestation

### General overview

In the early stage of syphilis, known as primary syphilis, a firm induration develops at the site of pathogen entry approximately 10 days to 3 months after infection. This subsequently evolves into a painless ulcer accompanied by regional lymphadenopathy. The affected areas are usually mucous membranes or adjacent tissues. Seroconversion usually occurs 14 to 21 days after exposure to the pathogen, although it may occur even later in individual cases. Initially, only IgM antibodies are detectable in serum, followed shortly thereafter by IgG antibodies. After 4 to 6 weeks, the primary lesion heals spontaneously. In 60–70% of patients, the primary lesion remains the only manifestation of syphilis [15].

During the phase of hematogenous and lymphatic dissemination of the pathogen, the disease is referred to as secondary syphilis. Symptoms are highly variable and may include fever, fatigue, headache, joint pain, and muscle pain [2, 16]. A characteristic presentation is a measles-like rash that initially predominates on the trunk and involves the palms and soles without itching. Virtually, any organ system may be affected during this phase, and involvement of the central nervous system (CNS) is not uncommon [16]. Studies using the rabbit inoculation test (RIT) demonstrated the presence of *Treponema pallidum* in the cerebrospinal fluid (CSF) of approximately 30% of patients with secondary syphilis [17]. Asymptomatic CSF pleocytosis was found in approximately 40% of patients. Since only 5–10% of untreated individuals later develop neurosyphilis years or decades after infection, spontaneous clearance of the pathogen from the CNS or failure of reactivation appears possible [18, 19]. Co-infection with HIV significantly increases the likelihood of neurosyphilis, suggesting impaired spontaneous pathogen elimination in HIV-positive patients. Symptoms of secondary syphilis generally resolve spontaneously after 3–12 weeks.

In the tertiary stage, which develops years after infection, the following manifestations may occur: nodular skin lesions, ulcerating granulomatous lesions (gummas), and cardiovascular complications such as syphilitic mesaortitis and aneurysms. At this stage, even high-dose antibiotic therapy results only in partial recovery because long-standing inflammatory processes regress only slowly and patients with paralytic or meningovascular neurosyphilis often develop small demyelinating lesions and senile plaques [18, 20, 21].

### Neurosyphilis

The clinical manifestations of neurosyphilis are highly variable. A distinction is made between early neurosyphilis with syphilitic meningitis and meningovascular neurosyphilis (and sometime syphilitic gummas) and late neurosyphilis with tabetic neurosyphilis (tabes dorsalis), paralytic neurosyphilis (general paresis), and/or syphilitic gummas [22].

#### Early neurosyphilis

**Syphilitic meningitis**

Syphilitic meningitis usually occurs during secondary syphilis within the first year after infection, although a later onset is possible. Typical features include headache, meningism, nausea and vomiting, and cranial nerve (CN) palsies. Commonly affected cranial nerves include CN VIII (vestibulocochlear nerve), CN VII (facial nerve), and CN III (oculomotor nerve). The optic nerve may also be involved. Additional manifestations may include radicular symptoms and vascular brainstem syndromes.

**Meningovascular neurosyphilis**

Meningovascular neurosyphilis combines meningeal inflammation and syphilitic vasculitis. Throughout the literature, it may be classified as either early or late neurosyphilis because it can occur months or even years after infection. Today it represents one of the most common manifestations of neurosyphilis. Patients with meningovascular neurosyphilis have an increased risk of stroke. Clinical manifestations are extremely heterogeneous (“the great imitator”) and depend on the location of vascular involvement.

The underlying pathology is usually an obliterative endarteritis affecting medium-sized arteries at the base of the brain, particularly the middle cerebral artery (MCA) and branches of the basilar artery [23, 24]. Syphilitic aortitis may also cause embolic infarctions. Neurosyphilis should therefore be considered a differential diagnosis in young stroke patients, especially men, who lack conventional vascular risk factors.

**Syphilitic gummas**

Syphilitic gummas are rare localized granulomatous mass lesions. They usually originate from the meninges over the cerebral convexities. Depending on their location, they may be clinically silent or associated with focal neurological deficits, hydrocephalus or epileptic seizures [25–27]. Multiple lesions are referred to as gummatous neurosyphilis. Like meningovascular neurosyphilis, gummas may occur in both early and late disease stages.

**Syphilitic myelitis**

Syphilitic myelitis has also been described and may present with partial or complete spinal cord syndromes. The meningeal component may manifest as headache, cranial nerve lesions, optic nerve damage, and rarely hydrocephalus.

#### Late neurosyphilis

**Tabetic neurosyphilis (tabes dorsalis)**

Tabetic neurosyphilis (*tabes dorsalis*) is a chronic progressive inflammation of the dorsal root ganglia and posterior nerve roots. Characteristic findings include loss of deep tendon reflexes in the lower extremities, impaired vibration sense (pallanesthesia), pupillary abnormalities, gait ataxia, hyperextension of the knee and hip joints, bladder dysfunction due to deafferentation, and optic nerve damage. A classic pupillary abnormality is the Argyll Robertson pupil, in which the pupils accommodate but do not react appropriately to light. Patients frequently complain of sudden, severe, lightning-like pains radiating into the legs or abdomen. These painful episodes are called tabetic crises.

**Paralytic neurosyphilis**

Paralytic neurosyphilis (*general paresis*, *dementia paralytica*, *progressive paralysis*) represents a form of chronic progressive encephalitis. Typical manifestations include progressive cognitive impairment, reduced judgment and insight, psychiatric symptoms, psychotic episodes, speech disturbances, headache, dizziness, abnormal pupillary reactions (commonly Argyll Robertson pupils), tremor of the tongue, facial muscle tremors, epileptic seizures, and reflex abnormalities. As the disease progresses, patients may develop severe dementia, urinary incontinence, fecal incontinence, and cachexia (marasmus) [28].

#### Asymptomatic (latent) neurosyphilis

Asymptomatic neurosyphilis is diagnosed when all of the following are present: positive syphilis serology, evidence of intrathecal production of treponemal antibodies, lymphocytic CSF pleocytosis and/or elevated CSF protein, and/or a positive CSF - Venereal Disease Research Laboratory (VDRL) test but neurological symptoms are absent. In such cases, alternative causes of CSF pleocytosis need to be excluded.

## Diagnosis

### Overview of diagnostic tests

Several diagnostic methods are available for the diagnosis of syphilis. These can be divided into direct and indirect (serologic) detection of the pathogen.

**Direct detection**

Direct identification of *Treponema pallidum* is possible by dark-field microscopy or polymerase chain reaction (PCR). However, because pathogen concentrations in cerebrospinal fluid are usually very low in neurosyphilis, these techniques are of limited value. Current evidence does not support routine PCR testing of CSF for the diagnosis or exclusion of neurosyphilis because published studies have demonstrated a low sensitivity (approximately 31.7–63.7%) and variable specificity (approximately 39.9–98%) [29, 30].

**Indirect detection: serologic testing**

Serological tests are divided into (i) non-treponemal and (ii) treponemal antibody tests. The (i) non-treponemal tests include the VDRLtest and the Rapid Plasma Reagin test (RPR). Both detect antibodies against cardiolipin. Cardiolipin is present not only in *Treponema* cell membranes but also in mitochondria of human, animal, and plant cells. Consequently, positive results correlate with inflammatory activity, but specificity is limited and false-positive reactions may occur. The (ii) treponema-specific antibody tests detect antibodies directed specifically against *Treponema pallidum*. Available methods include chemiluminescence immunoassays (CIA), Treponema pallidum hemagglutination assay (TPHA), Treponema pallidum latex agglutination assay (TPLA), Fluorescent treponemal antibody absorption test (FTA-ABS), Enzyme immunoassays (EIA), and immunoblots. Cross-reactivity is rare but may occur with non-venereal treponemal infections and with infections due to other spirochetes, particularly *Borrelia* species.

The FTA-ABS test is an indirect immunofluorescence assay detecting both IgG and IgM antibodies. In early syphilis, sensitivity reaches 82–90% only whereas it is as high as 96–100% in late syphilis [31]. Specificity ranges from approximately 94.5 to 98.6%. False-positive results may occur in patients with borreliosis with high antibody titers and in patients with systemic lupus erythematosus or other autoimmune disorders. Today, monovalent FTA-ABS assays are used to specifically detect IgG or IgM antibodies.

The 19 S-IgM FTA-ABS assay separates 19 S-IgM fractions from 7 S-IgG fractions before analysis [32]. This improves diagnostic accuracy because it reduces false-positive results (e.g., caused by rheumatoid factor) and false-negative results due to IgG-mediated antigen blocking.

When screening tests are positive, antibody titers are quantified. Treponemal IgM assays have a near-100% specificity and high sensitivity across all stages of infection [31]. A positive treponemal IgM in a previously untreated patient indicates an infection that requires treatment. Although total treponemal antibody tests are highly sensitive, they provide little information about current disease activity. This is because antibodies may remain detectable for life after successful treatment or spontaneous resolution. This persistent serological positivity is termed a so-called serologic scar. Furthermore, in very early syphilis or neurosyphilis, treponemal antibodies may still be negative [22].

**Diagnostic challenges in neurosyphilis**

Neurosyphilis remains difficult to diagnose because no single test is sufficiently sensitive and specific. In many English-speaking countries, active neurosyphilis is diagnosed on the basis of the following constellations [33, 34]: Reactive CSF-VDRL or -RPR in combination with elevated CSF white blood cell count (> 5 cells/µL) and/or elevated CSF protein (> 45 mg/dL). The specificity of CSF-VDRL and -RPR is high, especially in late neurosyphilis (0,95% CI VDRL 0,86 − 0,98; RPR 0,61 − 0,99) [32, 35]. However, sensitivity is limited: VDRL reaches a sensitivity of approximately 30 to 86% and -RPR of approximately 71% [35–37]. Therefore, a negative CSF-VDRL or -RPR does not exclude neurosyphilis. Additionally, neither test has officially been approved for CSF diagnostics in most countries, including Germany.

### Diagnostic tests

**Treponema pallidum particle agglutination assay (TPPA)**

Because of its high sensitivity, the *Treponema pallidum* Particle Agglutination Assay (TPPA) was long considered the gold standard in Germany for detecting intrathecal treponemal antibody production in suspected neurosyphilis. Following the unavailability of TPPA (the test is not distributed any more in many regions), only a limited number of alternative systems remain for calculating antibody indices. Their exact sensitivity and specificity have not yet been fully established. Available alternatives include TPHA-based methods and enzyme immunoassays (EIA). These tests appear to be less sensitive than TPPA, but they currently represent the only practical screening methods for CSF evaluation. Consequently, repeat testing may be indicated when clinical suspicion remains high despite initial non-reactive findings.

Evidence of intrathecal synthesis of treponemal antibodies can be determined using various calculation methods [38]. The most widely used approach is the *Treponema pallidum* Antibody Index (ITpA Index), which compares CSF and serum antibody concentrations. A crucial aspect of all calculations is correction for blood–CSF barrier function by relating treponemal antibody quotients to total IgG quotients [38, 39]. This accounts for matrix effects, blood–brain barrier dysfunction, and passive antibody transfer from serum.

It is important to keep in mind that treponemal antibody titers are not suitable as general markers of disease activity. Even after successful treatment, intrathecal treponemal antibody synthesis may persist for many years or even decades [22]. Clinicians should also be aware that borreliosis can occasionally produce false-positive treponemal antibody results.

**CXCL13 as a CSF biomarker**

**Fig. 1:**
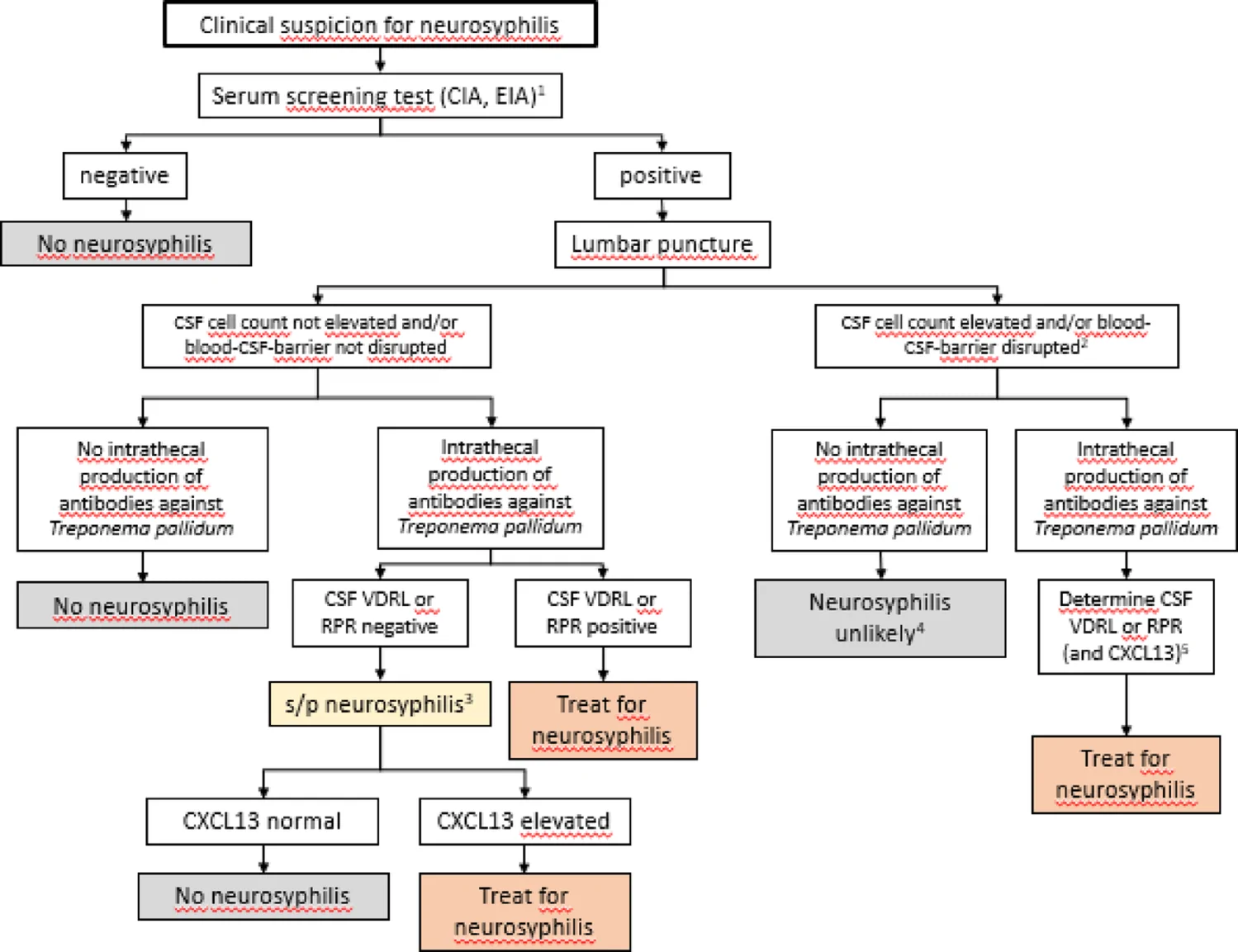
(1) A confirmatory test is recommended (see text). (2) In patients with HIV, CSF pleocytosis can be due to HIV itself. (3) If neurosyphilis is still considered, treatment should be taken into consideration. (4) In a very early stage of neurosyphilis, an intrathecal antibody production may not be detectable yet. (5) Determination of VDRL or RPR and possibly CXCL13 is recommended as a basis for follow up investigations. It has to be kept in mind that CXCL13 can also be elevated in other diagnosis. For details see text. CIA = chemiluminescence assay, EIA = enzyme immuno essay, VDRL = veneral disease research laboratory, RPR = rapid-plasma-reagin-test

Another important CSF marker is the chemokine CXCL13. Studies have demonstrated significantly elevated CSF CXCL13 concentrations in most (but not all) patients with neurosyphilis [40, 41]. Potential advantages include supportive diagnostic information, assessment of disease activity, and monitoring treatment response. However, several limitations exist: Not all neurosyphilis patients exhibit elevated CXCL13 levels, no consistent difference has been demonstrated between symptomatic and asymptomatic neurosyphilis, and elevated CXCL13 is not specific for neurosyphilis. Other disorders associated with elevated CXCL13 include neuroborreliosis, central nervous system lymphoma, and other B-cell-mediated inflammatory diseases [42]. Therefore, the sensitivity and specificity of CXCL13 remain incompletely defined. One important feature is that CXCL13 concentrations often decline rapidly following successful treatment, making it potentially useful for follow-up assessments [43, 44]. For this reason, the guideline recommends considering CXCL13 as an adjunctive marker when acute neurosyphilis is suspected (Fig. 1).

### Diagnostic criteria

Because of the limitations of non-treponema-specific tests, modified diagnostic criteria have been adopted in German-speaking countries. The diagnosis is based on a combination of (i) clinical findings, (ii) CSF findings, and (iii) evidence of intrathecal treponemal antibody synthesis. A distinction is made between probable neurosyphilis and definite neurosyphilis [22, 45].

**A. Probable neurosyphilis**

A patient is considered to have probable neurosyphilis if criterion (i) is fulfilled, and at least two of criteria (ii)–(iv) are present.

(i) Positive treponemal antibody screening test in serum incl. confirmatory testing.
(ii) Subacute or chronic progressive neurological and/or psychiatric symptoms with episodes of deterioration and partial remission.
(iii) Abnormal CSF findings, including mixed-cell or mononuclear pleocytosis and/or blood–CSF barrier dysfunction.
(iv) Clinical and/or CSF improvement after treatment with antibiotics recommended for neurosyphilis.

**B. Definite neurosyphilis**

Definite neurosyphilis is diagnosed when criteria for probable neurosyphilis are fulfilled, CSF pleocytosis and/or blood–CSF barrier dysfunction are present, and intrathecal synthesis of treponemal antibodies is found. If CSF-VDRL or CSF-RPR is also positive, the diagnosis is confirmed. However, it needs to be kept in mind that both CSF-VDRL and -RPR have limited sensitivity and may remain negative even in clinically relevant neurosyphilis, particularly in late-stage disease [22, 32].

### Diagnostic approach in patients with suspected neurosyphilis

**CSF findings**

The most common abnormalities are CSF pleocytosis and/or blood–CSF barrier dysfunction which often manifests as elevated CSF protein levels. Studies have reported pleocytosis in 82.7% and/or elevated protein in 68.1% in symptomatic neurosyphilis. In asymptomatic neurosyphilis, pleocytosis is present in 81,2% and elevated protein is found in 28.6% [46].

**Step 1: Serum screening**

When neurosyphilis is suspected clinically, the first step is a treponemal screening test in serum (Fig. 1). Preferred screening methods include chemiluminescence immunoassay (CIA) and Enzyme immunoassay (EIA). Positive screening results should be confirmed by FTA-ABS, ELISA, or Immunoblot.

**Step 2: Paired CSF and serum examination**

If serum treponemal antibodies are detected and neurosyphilis remains clinically possible, a paired CSF-serum analysis should be performed. The following investigations are recommended:

#### Basic CSF studies

Cell count, total protein, lactate, and CSF/serum glucose ratio (if available).

#### Further CSF analysis

Albumin quotient, IgG quotient, IgA quotient, and IgM quotient to evaluate blood–CSF barrier function and intrathecal immunoglobulin synthesis.

#### Specific neurosyphilis testing

Treponemal antibody index (see above for details), CSF RPR or VDRL, and optionally CXCL13.

**Special considerations in HIV infected patients**

Interpretation becomes more difficult in HIV-positive patients. Among HIV-positive patients with neurosyphilis pleocytosis was observed in only 58.7% and elevated CSF protein occurred in 53.3% [47]. Furthermore, HIV infection itself may cause mild pleocytosis and/or mild blood–CSF barrier dysfunction [2]. Therefore, isolated mild abnormalities are less specific in HIV-positive individuals.

**Role of the antibody index**

Demonstration of intrathecal treponemal antibody production via antibody index calculation is one of the most important pieces of evidence supporting neurosyphilis. However, clinicians should remember that antibody indices may still be normal very early in the course of the disease.

**Key recommendations**

**Recommendation**:

Patients with clinical suspicion of neurosyphilis should undergo serum syphilis screening.

**Recommendation**:

Patients with positive syphilis serology and neurological or psychiatric symptoms should undergo CSF examination.

**Recommendation**:

If CSF pleocytosis and/or blood–CSF barrier dysfunction is present, intrathecal synthesis of treponemal antibodies should be assessed.

### Interpretation of test results when CSF white cell and protein are normal

**Interpretation of isolated intrathecal antibody production**

A particularly challenging situation arises when intrathecal treponemal antibody production is detected while all other CSF parameters remain normal (no pleocytosis, no elevation of CSF protein, and no evidence of blood–CSF barrier dysfunction). In such cases, it is often unclear whether the finding represents a serologic scar resulting from a previous, resolved, or successfully treated neurosyphilis infection or if it is evidence for an active infection requiring treatment. If intrathecal antibody production is accompanied by a positive CSF VDRL, or a positive CSF RPR, treatment like in confirmed neurosyphilis may be considered [2]. Similarly, treatment may be justified when intrathecal antibody production is present and no alternative explanation exists for the neurological symptoms and CSF CXCL13 levels are elevated even if classical inflammatory CSF findings are absent.

**Recommendation**

When intrathecal antibody production against *Treponema pallidum* is detected without pleocytosis or blood–CSF barrier dysfunction, clinicians should consider the possibility of a serologic scar.

**Baseline studies before treatment**

If treatment for neurosyphilis is indicated, baseline investigations should include treponemal IgM antibodies and VDRL or RPR titers in serum and VDRL or RPR titers and optional CXCL13 measurement in CSF (Fig. 1). These baseline values may later help assess treatment response if clinical improvement is incomplete or uncertain.

**Recommendation**

In patients diagnosed with neurosyphilis, serum and CSF VDRL or RPR titers should be determined to establish a baseline for potential future follow-up assessments.

Measurement of CSF CXCL13 may additionally be considered.

### Evaluation of suspected latent asymptomatic neurosyphilis

An important clinical question is whether every patient with positive treponemal serology should undergo lumbar puncture to exclude asymptomatic neurosyphilis. This question most commonly arises when syphilis serology is found to be positive but no obvious neurological symptoms are present. Lumbar puncture should be performed in the following situations even when neurological symptoms are absent: (i) Clinical evidence of cardiovascular manifestations such as syphilitic aortitis or syphilitic aneurysm formation, (ii) Patients who worsen despite guideline-concordant treatment, (iii) When non-treponemal antibody titers (RPR or VDRL) fail to decline appropriately after treatment.

Historically, lumbar puncture was routinely recommended in patients with ocular manifestations such as papillitis or uveitis and otologic manifestations such as hearing impairment or vestibular dysfunction. Current evidence and CDC recommendations have changed this practice. Because ocular and otologic syphilis are treated using the same regimen as neurosyphilis, routine lumbar puncture is no longer recommended in the following constellation:

- No neurological symptoms are present,
- No cranial nerve involvement exists, and.
- No psychiatric manifestations are present.

**Recommendation**

Patients with isolated ocular or otologic manifestations of syphilis should not routinely undergo lumbar puncture.

**HIV infection and asymptomatic neurosyphilis**

Patients with HIV have an increased risk of latent asymptomatic neurosyphilis. Risk factors include absence of antiretroviral therapy, high HIV viral load, and/or low CD4 cell count [47, 48]. Several studies found that most HIV-positive patients with asymptomatic neurosyphilis had CD4 counts below 200 cells/µL [49]. Therefore, routine lumbar puncture in all HIV-positive patients with syphilis is not recommended anymore. However, CSF examination may be considered when CD4 count < 200 cells/µL, Serum VDRL > 1:64, or markedly elevated RPR titers [45].

Interpretation of mild CSF abnormalities is difficult in HIV-positive patients because HIV infection alone may cause mild pleocytosis and/or mild blood–CSF barrier dysfunction. Therefore, findings must be interpreted cautiously. Treatment for neurosyphilis in HIV patients is generally recommended when intrathecal treponemal antibody production is present and CSF VDRL is positive. CXCL13 measurement may provide additional support for decision-making.

### Additional diagnostic evaluation

**Neuroimaging**

All patients with neurosyphilis should undergo brain imaging. The preferred modality is Magnetic Resonance Imaging (MRI). Depending on the clinical presentation, further investigations may include neurophysiological studies [electroencephalography (EEG)], imaging (spinal MRI, transcranial doppler sonography, plain radiography when indicated) and/or specialist evaluation (ophtalmologic, otologic, neurourological).

**Recommendation**

Patients with neurosyphilis should undergo MRI of the brain and, when clinically indicated, MRI of the spinal axis.

Because syphilis is a sexually transmitted infection, newly diagnosed patients should undergo screening for additional infections. Recommended tests include HIV testing, hepatitis B, and hepatitis C testing. Further sexually transmitted infection (STI) screening should follow current STI guidelines.

### Differential diagnosis

The differential diagnosis depends on the stage and clinical presentation of disease. Particularly in cases of probable rather than definite neurosyphilis, a broad differential diagnostic evaluation is required. Potential mimics include numerous neurological, psychiatric, infectious, inflammatory, autoimmune, and vascular disorders. Because of the large number of possible differential diagnoses, the guideline does not discuss individual diseases in detail.

## Treatment

### Antibiotic therapy

Antibiotic treatment is indicated in all stages of neurosyphilis, ocular syphilis, and otosyphilis [2, 34, 45, 50]. High-dose Penicillin G has been associated with an increased risk of seizures [51]. Patients with a history of epilepsy or previous seizures should therefore receive appropriate antiseizure therapy during treatment. An accepted alternative is Ceftriaxone [22, 34, 52]. The authors note that no convincing evidence supports an initial loading dose of 4 g, which had been suggested in older guideline versions.

**Recommendations**

Patients with neurosyphilis should receive intravenous penicillin G or intravenous ceftriaxone.

The recommended dosage of penicillin G is 6 million IU IV every 6 h, or 5 million IU IV five times daily, or 10 million IU IV every 8 h.

The recommended dosage of ceftriaxon is 2 g daily.

Recommended duration: 14 days.

When both penicillin G and ceftriaxone are contraindicated, treatment with doxycycline may be considered in adult patients. The rationale for this recommendation includes proven efficacy of doxycycline in systemic syphilis, adequate penetration into cerebrospinal fluid at this dosage, and several published case reports documenting successful treatment of neurosyphilis [2, 53]. However, important limitations exist - no randomized controlled trials are available and neither the CDC nor current European guidelines formally recommend doxycycline as standard therapy for neurosyphilis [52, 54]. Doxycycline should not be used in pregnancy and children younger than 8 years of age because of the risk of permanent tooth discoloration.

**Recommendations**

In adults with neurosyphilis who cannot receive penicillin G or ceftriaxone and in whom pregnancy has been excluded, doxycycline therapy may be considered.

Recommended dosage: 200 mg twice daily.

Recommended duration: 28 days.

### Management of complications

**Jarisch–herxheimer reaction**

One of the most important complications of neurosyphilis treatment is the Jarisch–Herxheimer reaction. This reaction should be suspected when symptoms occur approximately 12–24 h after initiation of antibiotic therapy. Possible manifestations include (i) general symptoms such as fever, headache, muscle pain, malaise, and fatigue, (ii) cardiovascular findings such as tachycardia, elevated blood pressure, and hypotension, (iii) laboratory findings such as leukocytosis and relative lymphopenia, and (iv) neurological manifestations such as seizures and worsening neurological deficits. Jarisch–Herxheimer reactions are common during secondary syphilis but occur in only approximately 1–2% of patients treated for neurosyphilis. Affected patients should receive careful cardiovascular monitoring and symptomatic treatment. Possible treatments include nonsteroidal anti-inflammatory drugs (NSAIDs) and corticosteroids when clinically necessary [55, 56]. Although corticosteroids are frequently discussed in case reports, there is currently no convincing evidence supporting routine adjunctive corticosteroid therapy for neurosyphilis itself or prevention or treatment of a Jarisch–Herxheimer reaction. In case of a Jarisch-Herxheimer reaction, antibiotic therapy should not be interrupted.

## Follow-up and assessment of treatment response clinical assessment

The primary measure of treatment success is clinical improvement. When a patient demonstrates a satisfactory clinical response, a routine repeat lumbar puncture is not required [22, 52].

**Serologic follow-up and VDRL RPR monitoring**

Monitoring of serum treponemal IgM antibodies may be helpful. Typical findings include declining IgM titers within 6–12 months and even disappearance of treponemal IgM antibodies within approximately 18 months. However, reinfection and/or delayed initiation of therapy may prolong persistence of IgM antibodies. Following successful treatment, CSF VDRL or RPR titers often decline by 3–4 dilution steps during the first year and complete conversion to a negative VDRL or RPR is considered strong evidence of treatment success [22, 52]. Nonetheless, non-treponemal antibodies may remain detectable longer in the case of reinfections and long-standing infections even after adequate therapy.

Tests Unsuitable for Follow-Up.

The following tests are not useful for monitoring treatment response: TPPA, FTA-ABS IgG, Intrathecal treponemal IgG synthesis as these markers frequently remain positive despite successful treatment.

**Necessity of repeated CSF investigation**

Repeat CSF examination 4–6 months after therapy (or earlier) should be considered when clinical improvement is absent, clinical improvement is insufficient or doxycycline or another inadequately validated regimen was used. Early reassessment may be warranted if neurological symptoms worsen. Distinguishing relapse from reinfection may be difficult. A relapse should be considered in worsening of pre-existing neurological symptoms after treatment. Reinfection should be taken into account in case of new clinical signs of early syphilis and/or evidence of recent exposure. However, new neurological manifestations can occur in both situations.

**Recommendations**

Treatment response in neurosyphilis should primarily be assessed through clinical follow-up.

Repeat CSF examination may be considered after 4–6 months.

CSF reassessment is particularly recommended when clinical response is inadequate and/or non-standard antibiotic regimens have been used.

**Indicators for re-evaluation**

Further diagnostic work-up and possible retreatment should be considered when pleocytosis fails to improve within 6 months or elevated CSF cell counts persist for 2 years or a fourfold increase in treponemal or non-treponemal titers. In HIV-positive patients, normalization of CSF abnormalities may occur more slowly. Persistent pleocytosis despite adequate treatment should prompt evaluation for alternative diagnoses.

## Prognosis

Historically, syphilis was a major cause of mortality. In 1968, 586 deaths from syphilis were recorded in the United States. Between 1998 and 2015, annual deaths ranged from only 24 to 46 cases [57]. The prognosis of neurosyphilis has improved substantially because of effective antibiotic therapy. Despite successful treatment, residual neurological deficits are common. In a retrospective study of 142 patients with neurosyphilis, 41.8% developed permanent sequelae. Reported residual deficits included cognitive impairment (28.8%), upper motor neuron signs (18.6%), seizures (16.9%), ataxia (16.9%), cranial nerve palsies (15.2%), visual impairment (11.8%), gait disturbances (6.7%), aphasia (6.7%), and hemiparesis/hemiplegia (6.7%) [58]. Thus, despite modern treatment, neurosyphilis remains a disease with substantial potential for long-term neurological disability.

## Data Availability

No datasets were generated or analysed during the current study.
